# Plant-Based Cheeses: A Systematic Review of Sensory Evaluation Studies and Strategies to Increase Consumer Acceptance

**DOI:** 10.3390/foods10040725

**Published:** 2021-03-30

**Authors:** Erin C. Short, Amanda J. Kinchla, Alissa A. Nolden

**Affiliations:** Department of Food Science, University of Massachusetts, Amherst, MA 01003, USA; ecshort@umass.edu (E.C.S.); amanda.kinchla@foodsci.umass.edu (A.J.K.)

**Keywords:** non-dairy cheese, soy-based cheese, sensory evaluation, consumer acceptance

## Abstract

Animal protein provides unique sensory and textural properties to foods that are not easily replicated when replaced with plant-based alternatives. Food scientists and researchers are currently developing innovative approaches to improve their physical and sensory characteristics in plant-based analogs. In terms of plant-based cheese substitutes (PBCS), soy is the most commonly used plant-based protein but is associated with undesirable sensory attributes (i.e., beany and gritty). In order to determine if the approaches result in a significant improvement in sensory quality and liking, sensory evaluation is employed. The aim of this review is to summarize the original literature (*n* = 12) relating to 100% PBCS which utilizes sensory evaluation methods. Overall, a major theme identified in this review is the innovative strategies used to increase acceptance of PBCS, whether products are aimed at improving existing non-dairy-based cheese formulations or to more closely mimic a conventional dairy-based cheese product. Studies demonstrate processing and fermentation of soybeans and blending of non-dairy milks are potential ways to improve consumer liking of PBCS. A secondary focus is to discuss the current sensory methodology carried out in the reviewed literature. Future studies should consider using more specific measures of flavor and mouthfeel, integrate evaluation of consumer liking with instrumental textural methods, and use a larger more diverse group of consumers. The outcome of this review is to highlight the importance of integrating sensory science in order to help facilitate the improvement of the sensory and quality attributes of PBCS and streamline product development.

## 1. Introduction

Augmented interest in plant-based foods has increased due to concerns related to health, sustainability, and animal welfare. In terms of conventional production of dairy, there are three major areas of concern: environment impact (emissions of greenhouse gases, pollution of soil and water, and land use), human health (exposure to zoonotic diseases and increased antibiotic resistance), and animal welfare (treatment of farmed animals, including disease, injury, and mental/emotional well-being) [1]. Therefore, plant-based products offer a more sustainable and ethical option to consumers that are rapidly increasing in popularity among consumers. As a testament to this, U.S. retail sales of plant-based substitutes that directly replace animal products grew 29% between 2017 and 2019 to reach a USD 5 billion market. In the dairy category plant-based cheese substitutes (PBCS) saw the most growth in a year-over-year retail sales comparison by increasing 95% in 2020 [2,3]. Within the plant-based product market, PBCS is an emerging segment that has yet to gain traction or interest from a diverse consumer base [4]. Although PBCS sales continue to grow, the category remains in its infancy compared to other plant-based analog categories (i.e., dairy and meat) [5,6] as PBCS only accounts for less than 1% of all total dollar sales of retail cheese [3]. In order to increase acceptance of these products, sensory methods can be employed to better understand sensory and quality attributes and whether they provide the desirable qualities of a conventional dairy-based product.

Consumer liking is arguably the biggest challenge for any plant-based substitute. From a US consumer standpoint, only 32% agree that PBCS taste as good as regular cheese, while 34% disagree, and another third of cheese consumers remain indifferent [4]. If the PBCS express quality attributes that meet consumers’ expectations, then these substitutes can be a satisfactory replacement for a dairy counterpart [4]. The Food and Health Survey consistently shows that taste is the number one driver behind purchase intention [7], more important than price, convenience, and health. Therefore, it is important to develop a product that provides desirable sensory characteristics (i.e., taste, flavor, and mouthfeel properties). In summary, the low sales margins indicate that the consumer market is left unsatisfied and there is substantial room for improvement and growth within this product category.

For PBCS, there are two general approaches to the flavor profile: (1) those which intend to mimic the sensory attributes of conventional dairy cheese and (2) those that embrace the unique flavors and characteristics derived from the plant. The challenge with the former is that plant-based ingredients do not precisely mimic the sensory (i.e., flavor, taste, and aroma) and physical (i.e., mouthfeel and meltability) characteristics of dairy-based cheese which limits consumer acceptability. Yet, the latter expresses novel characteristics in products that may not be desirable to the majority of consumers which has resulted in a specific and narrow niche market. Regardless, if the goal is to mimic or embrace the sensory characteristics, the end product should have physical, functional, and sensory properties that consumers find desirable [8]. To achieve this, sensory evaluation, specifically hedonic evaluation, must be employed to assess the product performance.

Among the accessible published literature of PBCS, the focus has been on soy-based “spreadable” products. This work is not representative of the diverse ingredients used in PBCS products displayed on grocery store shelves. Between 2015 and 2020 coconut oil was the top ingredient in new PBCS, with other common ingredients dominating the retail market include modified and native starches and nut milks [9,10], while available research tends to focus on soft “spreadable” soy-based products. Soymilk’s functional and nutritionally complete proteins [11] are comparable to cow’s milk and the availability and affordability of soy [6] are some reasons why researchers tend to focus more on this dairy alternative. One potential limitation of soybeans are the concerns with high estrogen levels [12] and being a common food allergen. Relying on plant-based ingredients to simulate the processed nuance of dairy cheese, including the physical, chemical, and functionality properties, is no easy task and sensory challenges are inevitable. Like any plant-based ingredient, soybeans in particular exhibit flavor and mouthfeel challenges, specifically, beany flavor and gritty mouthfeel [6].

To the authors’ knowledge, there has not been a review of the sensory attributes of PBCS. This systematic review focuses on the studies that have conducted sensory evaluation of PBCS. After performing the search, (see Methods below), it became clear that all but one of the studies focused on soy-based products and applied different processing techniques, approaches, or methods to improve consumer liking. As a result, the goal of this review is to summarize the literature aimed at improving consumer liking of soy-based PBCS. This review is organized by first summarizing the breadth of work published that aims to improve the sensory attributes and describe the results of the consumer sensory evaluation. We then summarize the current limitations within the existing literature in terms of the sensory evaluation methodology and suggest several areas of needed exploration that have yet to be examined (i.e., consumer segmentation and alternative plant-based proteins). There is an opportunity to improve the PBCS market by decreasing undesirable sensory characteristics and improving overall liking. In order to achieve this, sensory science should complement ongoing scientific research regarding PBCS in order to directly improve the quality and sensory attributes and lead to more efficient development processes.

## 2. Methods and Search Criteria

A systematic search was carried out using the Web of Science and Google Scholar on studies published in English through March 2021. The search used keywords pertaining to plant-based and imitation cheeses and included sensory evaluation. The following keywords included: “cheese analog(ue)s”, “cheese substitutes”, “imitation cheese”, “cheese replacement”, “alternative cheese”, “non-dairy cheese”, “cheese-like” and “plant-based cheese.” Consumer studies coupled with other sensory evaluation analytical methods were selected using “consumer liking”, “consumer acceptance”, “consumer perception”, “sensory quality”, “sensory characteristics”, “sensory properties”, “sensory evaluation”. The initial search identified 33 articles. We excluded online focus groups, surveys, and questionnaires, where data was collected based on verbal or visual information and not through sampling products (*n* = 8 articles). Following an initial search and secondary screening of the above criteria, the search resulted in the selection of 25 articles.

Within the plant-based literature, the term “analogue” is reserved for plant-based products that simulate properties of the conventional animal-based product [13]. While this established term indicates no animal-derived ingredients, within PBCS literature “analogue” is interchangeable for both 100% plant-based and products made with partial dairy ingredients [14,15]. This dual use of the term “analogue” in regard to PBCS has led to confusion within the scientific community. This review focuses on cheese analogs made without any dairy ingredients and studies involving 100% plant-based ingredients. Of the 25 articles found within our defined search query, we excluded 13 papers that evaluated cheese analogs made partially with dairy. Of the remaining 12 articles which evaluated 100% plant-based cheese: 10 PBCS were soft, spreadable products while 2 focused on hard or semi-hard cheeses; 5 were made with only soy and 6 evaluated blended ratios of soy and nut milks; 1 evaluated commercially available coconut-based cheese (Table 1). Only one study purchased commercially purchased products, all other studies created the product for the purpose of the study. Three studies included a conventional dairy-based product as a control [10,16,17]. All studies included a measure of acceptance or liking while four studies [10,16,18,19]. performed a combination of descriptive and hedonic evaluations (Table 1). It was also noted that several studies had limitations in their methodology for sensory testing, which are common errors, such as small participant pools, choice of control, and usage of trained panelists (described below in Section 4).

## 3. Literature Review

The sensory challenges for developing soy-based PBCS products have been most often attributed to undesirable beany flavor and gritty mouthfeel [6,16,27]. The beany flavor from soybeans occurs as a result of lipoxygenase activity [27], which does not occur in undamaged raw soybeans; however, in the presence of water and oxygen, an enzymatic process takes place and emphasizes off-flavors [28]. The gritty mouthfeel is a result of the larger, rough particulates, made up of proteinaceous, carbohydrate, and cellulosic components [29]. These attributes are often considered to be “off characteristics” which diminish the overall quality and acceptance of the soy-PBCS while their dairy counterparts remain smooth and uniform [16]. Adapting various soybean processing methods (i.e., posed solutions of fermenting, blending milks, blanching, and/or adding sodium bicarbonate) report a refinement in sensory characteristics which is necessary in order to increase overall liking.

One study completed a sensory profile of commercially available plant-based cheese and compared their acceptance to a conventional dairy-based cheese product. The remaining literature (*n* = 11) focuses on evaluating processing strategies to improve the liking and sensory qualities of PBCS (refer to Table 1). These strategies can be divided into three categories (1) modified fermentation (*n* = 3), (2) blending milks (*n* = 4), and (3) modified processing (*n* = 4) of soybeans. Here, we summarize the results of these strategies in terms of improving their liking using sensory evaluation methods, all of which suggest an improvement in sensory profile resulting in increased liking of PBCS. 

### 3.1. Strategies to Improve Consumer Liking

#### 3.1.1. Modified Fermentation

In order to prevent a gritty mouthfeel (sedimentation of large particles) and ultimately obtain a soy-PBCS with a smooth texture, alternate methods include the adoption of fermentation techniques. Specifically, the incorporation of lactic acid bacteria (LAB) softens the rough particulates in order to achieve a smoother texture when blended [30]. The role of soymilk fermentation is suggested to aid in removing the undesirable beany flavor while inadvertently improving the nutritional composition [16]. An additional method of incorporating sodium bicarbonate increases the pH which affects the protein structures of the soybean seed coats and allows for the reduction of the gritty mouthfeel [31]. The following studies incorporated combination approaches of sodium bicarbonate and various fermentation techniques were suggested to improve the beany and gritty characteristics expressed by soybeans, although no study specifically measured these characteristics.

Two studies Li, Q. et al. [16] and Li, Y. et al. [18] coupled both hedonic and descriptive testing methods to evaluate whether modified fermentation improved the sensory attributes and acceptance PBCS. Li, Y. et al. [18] initially soaked the soybeans in a 0.5% (wt/vol) sodium carbonate solution for 20 min before creating the soymilk. The milk was then inoculated with 3% of the LAB starter culture and/or *Geotrichum candidum* at 10^4^ CFU/mL before undergoing fermentation. The control PBCS sample prepared with the LAB starter culture (which was not inoculated with *G. candidum*) was stored at 4 °C and used to compare maturation differences between the samples prepared with the combination of LAB and *G. candidum*. The samples were ripened in a variety of temperatures (4 °C, 10 °C, and 15 °C) and assessed at three different aging durations (21, 28 and 35 days). In the hedonic test, 10 trained panelists rated liking using a 5-point scale (0 = inconsumable, 1 = unacceptable, 2 = acceptable, 3 = satisfactory, and 4 = excellent). The panelists indicated that the highest-rated PBCS in terms of color, flavor, appearance, and overall liking was the sample which combined the LAB and *G*. *candidum* approaches (ripened at 10 °C for 28 days). Panelist ratings fell within the “excellent” category, which was higher compared to the traditional LAB PBCS product, which ranked “satisfactory” in terms of color, flavor, appearance, and overall acceptability. Following the hedonic analysis, a descriptive test was performed with the same panelists using a 5-point intensity scale (1 = little to 5 = very much). Ratings were collected for hardness, springiness, and chewiness. In terms of textural profile, the authors described the textural attributes to be improved in the combination approach describing the product to change from “brittle and hard” to “soft and sticky”. The authors concluded that the combined approach exhibited a more stable, homogeneous structure, and presumably reduced the undesirable beany and gritty sensory properties, which ultimately increased the consumer likeability. It should be noted that the study did not ask panelists to rate the samples in terms of beany or gritty sensory attributes in order to determine whether this affected consumer acceptance. For this study, no formal statistics were reported for collected liking and intensity ratings. This study also performed objective measures of textural properties using a texture profile analyzer and measured the hardness, adhesiveness, springiness, cohesiveness, gumminess, chewiness, and resilience. While it can be advantageous to pair instrumental and sensory methodologies, in this case, no formal comparison was performed between instrumental and sensory data.

Using a similar approach as above, Li, Q. et al. [16] first soaked soybeans overnight in a 0.1% (*w*/*v*) sodium bicarbonate solution before undergoing different fermentation conditions which included LAB, glucono-delta-lactone coagulation, and/or enzymatic hydrolysis. Sensory analysis was used to identify which fermentation approach resulted in an increased consumer liking, and compared intensity ratings of appearance, color, creaminess, firmness, spread-ability, and flavor to a dairy-based control. Sensory characteristics were rated on a 9-point structured scale by 10 trained panelists. The results indicated that the highest-rated PBCS sample for all attributes was prepared using a combination of LAB and glucono-delta-lactone processes. This sample received an overall liking rating of 7.4 which was significantly different compared to other PBCS (only using one fermentation method), with ratings between 6.3 and 6.8. Although the dairy control ranked significantly higher in terms of overall liking, scoring a 7.7, the utilization of the combined fermentation methods made a significant improvement in the overall liking compared to PBCS products. The dairy-based control performed significantly better for every attribute, except for creaminess, which was not significantly different from PBCS prepared with combination of LAB and glucono-delta-lactone processes. Results of this study exemplify how a PBCS ranks in comparison to a conventional dairy cheese product and how incorporating a combined fermentation method can help improve the sensory characteristics of PBCS. Similarly, a texture profile analyzer was used to quantify textural properties (hardness, adhesiveness, springiness, cohesiveness, gumminess, chewiness, and resilience); however, these results were not compared to hedonic performance. Combining instrumental and sensory data can be useful to identify relationships between textural properties and consumer liking. Further, it is suggested that this approach increases liking as a result of reducing the perception of beany or gritty sensory attributes; however, these attributes were not directly measured in this study.

Chumchuere et al. [19] evaluated the physicochemical properties of a (fried vs. unfried) semi-hard soy PBCS which utilized a combined fermentation approach, inoculated with LAB and *Streptococcus thermophilus*, and was ripened at 4 °C for 7 days. A group of 14 participants used a linear scale to rate liking and intensity of sensory attributes in order to identify if frying improved the sensory characteristics and overall liking. Results of the hedonic test indicated that the fried sample received a significantly higher rating for overall liking (average rating 54.1) compared to the unfried sample (average rating 33.5). The participants rated the intensity of taste (acidity, salty, bitterness, and astringency), flavor (strong, cheesy, fermented, beany, rancid), texture (firmness and open texture), and color. There were significant differences between the unfried and fried PBCS for color, firmness, open texture, astringency, and all but one flavor attribute (strong, fermented, beany, and rancid). Sensations that were rated as more intense included color, firmness, open texture, and strong flavor, and reduced intensity ratings for astringency and fermented and beany flavor. This study was able to confirm that frying PBCS increases liking by reducing undesirable sensory attributes (astringency, beany and fermented flavor). Intensity ratings on quality and sensory attributes help to understand the change in characteristics as a result of modified processing, which led to an increased hedonic rating.

#### 3.1.2. Blending Milks

Blending different ratios of plant-based milks with soymilk results in an improved flavor profile compared to 100% soymilk PBCS. While no study specifically evaluated off-flavors or perception of beany flavor in any product, it is suggested that this attribute is reduced as a result of blending soymilk with alternative non-dairy milks. Adejuyitan et al. [20] created soft PBCS prepared using fermented soybeans prior to blending soy and coconut milks at 5 different ratios to combat the naturally beany flavor of soymilk. Using a 9-point hedonic scale, ratings from 10 untrained participants indicated that the highest-rated PBCS sample in terms of flavor, texture, taste, and mouthfeel, and overall acceptability was the 50:50 soy/coconut blend. This sample had an average overall acceptability rating of 7.3 compared to the 100% soy control, which was rated at 5.6, resulting in a significant difference between the two samples. This study shows that the combination of the two methodologies, fermentation and a blended ratio of plant-based milks, resulted in improved flavor, texture, and increased consumer liking.

Khodke et al. [21] created six soft PBCS at varying ratios of soy to groundnut (a legume crop formally known as peanut) milk in order to reduce the naturally occurring beany flavor of soymilk. Results of a hedonic evaluation (9-point hedonic scale) performed by 10 trained participants indicated the 90:10 soy/groundnut ratio received the highest acceptability rating compared to other ratio blends, including PBCS control, in terms of color, flavor, appearance, texture, and taste. When comparing the 90:10 blend to the PBCS control, flavor (7.8 vs. 6.3) and appearance (8.6 vs. 7) attributes saw the greatest improvement in ratings. The 90:10 blend received an average rating of 8.0 for overall acceptability while the control, 100% soy, rated 7.5. Statistical analysis was performed but was not structured in a way to evaluate whether the attributes were significantly different across sample categories. This comparison provides evidence that incorporating groundnut milk with soymilk can improve the flavor and texture which can help to increase overall acceptability.

Balogun et al. [22] created soft PBCS by blending soy and tiger nut milks prepared with six different ratios to combat the naturally beany flavor of soymilk. Using a 9-point hedonic scale, 20 untrained participants screened for PBCS product consumption indicated that the blended product prepared with 95:5 soy to tiger nut ratio received the highest ratings in terms of color, taste, texture, aroma, and overall acceptability. This blended PBCS had an average rating of 7.4 for overall acceptability where the 100% soy control had an average rating of 6.3. Statistical analysis revealed no significant difference between the 5% tiger nut blend and control PBCS. Significant differences were observed for liking of taste ratings between 5% tiger nut blend and control PBCS, with the blended product receiving significantly higher ratings (7.0 vs. 5.7, respectively). Even with a small percentage (5%) of soy milk replaced with nut milk, it is suggested to reduce the beaniness of soy which results in improved flavor, texture, and overall consumer liking. For this study, it was not determined whether beaniness was specifically reduced; however, it was observed to improve taste compared to a 100% soy PBCS. Even though the incorporation of tiger nut milk improved liking of taste, for this study, it did not translate to a significant increase in overall acceptability of PBCS.

Other than blending with nut milks, one study has provided evidence of blending soy with carrot puree to improve the liking of soy-based PBCS compared to a dairy control. Butool et al. [16] incorporated different ratios of carrot puree at 10% and 20% ratios in order to improve the appearance, flavor, texture, color, acceptance, and nutritional value of the soy-PBCS. Although off-flavors were not exclusively acknowledged by the authors, it was understood that soybeans naturally express beany flavors and that incorporating carrot puree within the PBCS may aid in masking these undesirable characteristics. The samples were compared to the customary dairy counterpart, 100% buffalo milk, which acted as the control, and all were prepared in a traditional curry dish. Participants included 5 trained and 15 semi-trained participants and were asked to rate each sample on a 9-point hedonic scale in terms of color, appearance, flavor, and overall liking. The sample with 20% carrot puree received the highest average rating of 8.4, compared to the dairy control sample which had an average rating of 8.5. A full-soy control was also rated by participants, receiving an average overall acceptance rating of 7.1. The greatest increase in ratings was observed for flavor, 6.5 compared to 8.3 for the PBCS control and 20% puree PBCS, respectively. While no formal statistics were performed, the authors concluded that incorporating carrot puree can lead to improvement in sensory characteristics (color, appearance, flavor, mouthfeel, taste, and overall liking). This study provides preliminary evidence that the incorporation of carrot puree into a soy-PBCS is able to produce a product that is not different from a conventional animal cheese product when incorporated in a meal.

#### 3.1.3. Modified Processing of Soybeans

In order to eliminate the beany flavor, blanching and grinding soybeans at or above 80 °C has shown to reduce lipoxygenase activity in order to improve these sensory properties [16]. Additionally, incorporating sodium bicarbonate results in the softening of soybean seed coats [31] in order to reduce the gritty mouthfeel expressed [6]. In the following studies, the methods of sodium bicarbonate, blanching, or blending a variety of plant-based milks at different ratios were utilized in order to improve the sensory characteristics of soy-based PBCS. This section reviews studies that performed modified processing, which often entails blending soybeans with other non-dairy milks.

Kadbhane et al. [23] created a spreadable PBCS with soybeans that were blanched in 0.5% NaHCO₃ solution for 10 min prior to blending 5 different ratios of soy to coconut (90:10, 80:20, 70:30, 60:40 and 50:50). All samples were ripened at 4 °C, sampled at day 1, day 3, and day 6 of maturation. For this study, there was no 100% soymilk PBCS control sample, and instead the samples were compared against each other and the maturation days prior. Results of a composite scoring hedonic test, with an unknown amount of participants, indicating the most preferred maturation period fell at day 1. In terms of PBCS samples, the 50:50 sample received the highest ratings in terms of appearance, texture, color, flavor, and overall acceptability. Overall, the 90:10 ratio consistently was rated the lowest while the 50:50 blended ratio was rated the highest in every category and for every maturation day (1, 3, and 6). For this study, no statistical analysis was performed. Nonetheless, this provides preliminary evidence that blending soy and nut milks and sodium bicarbonate could help to improve the likeability of PBCS.

James and colleagues [24] created 3 soft soy PBCS samples using different coagulants (lime juice, alum, and steep water) to further understand the physicochemical, sensory, and microbial effects of the PBCS. A hedonic test was performed with 20 participants who rated appearance, aroma, taste, mouthfeel, and overall acceptability on a 9-point hedonic scale. The results indicated that only liking of mouthfeel was perceived as significantly different, with lime coagulated PBCS receiving the highest rating and significantly higher than the steep water coagulated PBCS, with no difference between either sample to the alum coagulated PBCS. There was a trend for the lime coagulated PBCS to be rated higher for all other attributes, but this was not significant. It should be noted that off-flavors and gritty mouthfeel were not exclusively acknowledged by the authors. This study suggests that lime could be used as a coagulant in order to improve mouthfeel properties, and future studies may want to specifically evaluate the sensory characteristics to determine if this helps to reduce the undesirable gritty characteristic.

In contrast to the studies described above, the following two articles used the largest sample sizes, *n* = 30 and *n* = 50, respectively [25,26], and sensory remained the focal point of the articles. Oyeyinka et al. [25] created soft PBCS while utilizing techniques of blanching soybeans for 30 min prior to blending soy and cashew milks at six different ratios in order to combat the naturally beany flavor of soymilk. The results of a 9-point hedonic test performed with 30 untrained participants (screened for product usage) indicated that blending soy with cashew milk did not result in any significant differences in any measures of liking, including overall acceptability. For this study, it is suggested that the addition of cashews may provide nutritional benefits while having the same level of acceptance among consumers compared to full soy PBCS.

Arise et al. [26] created a soft spreadable PBCS using a fermentation process that combined different blends of soy and almond milk ratios, which was tested in a sensory experiment prepared as breaded and fried. Although beany flavor was not exclusively acknowledged or described as a challenge, it is understood that all soybeans naturally express a beany flavor, and frying the PBCS coupled with the techniques described above will aid in decreasing these naturally undesirable characteristics. Results of a 9-point hedonic test performed with 50 untrained participants screened for “regular cheese consumption” indicated that the fried 70:30 soy to almond milk PBCS sample received the highest ratings in terms of overall acceptability. The 70:30 blend resulted in a significant improvement in overall acceptability compared to the 100% soy control (7.6 vs. 7.0, respectively). However, when looking at liking ratings for taste, color, texture, and aroma there were no significant differences in ratings between the 70:30 blend and 100% soy PBCS, but there was a trend for the 70:30 blend to have higher ratings for taste, color, and texture. Additional sensory studies are needed to determine if this approach results in masking of beany flavors or reducing grittiness. Overall, this study suggests blending soy with almond milk can improve the overall acceptability of PBCS.

### 3.2. Sensory Profile of Coconut-Based Cheese Products

Saraco and Blaxland [10] aimed to investigate if PBCS products were able to mimic the physical, sensory, and functional properties of their dairy counterparts or if further improvement would be needed. In contrast to the articles reviewed above, the authors did not create a PBCS but rather employed a descriptive sensory evaluation to assess the product performances between commercially available dairy and non-dairy cheeses in the UK. It was found that of the 109 commercially available PBCS, 74% of these products had coconut oil as their primary ingredient, while only 3% were soy-based. The most abundant variety of PBCS was mild cheddar. Based on these findings, the PBCS products that underwent sensory evaluation were all coconut-based and of the mild cheddar and semi-hard Italian varieties. In the descriptive analysis performed by 1 semi-trained and 3 trained panelists, two mild cheddar PBCS and two semi-hard Italian PBCS varieties were compared to their dairy counterparts and assessed based on their appearance, color, odor, mouthfeel, flavor, and aftertaste. Panelists also reported whether the PBCS products were deemed acceptable compared to their conventional dairy cheese. It was noted that not all PBCS sensory attributes were considered simultaneously comparable to their dairy counterparts. Results of the descriptive analysis concluded that neither the texture or flavor expressed in the semi-hard Italian PBCS were regarded as acceptable. The “yeasty” and “unpleasant onion/garlic” flavors, “oily” mouthfeel, and “sour” aftertaste were deemed potentially unacceptable to consumers compared to the dairy counterpart. While one of the mild cheddar PBCS expressed acceptable texture, the “rancid (intense)” odor, and “intense cheese rind” flavor deemed this sample potentially unacceptable to consumers. The other mild cheddar PBCS sample was the only non-dairy sample to have potentially acceptable attributes. Although the mild cheddar PBCS sample did not have a typical texture found in cheese it was deemed acceptable in both flavor and texture and was described as the following: with a “glossy, cheese-like, smooth” appearance, “pale yellow” color (similar to dairy sample), “waxy/mild/parmesan-like” odor, “oily/rubbery” mouthfeel (less resistance compared to dairy sample), “intense/typical processed cheese-like” flavor, and a “salty” aftertaste. This study demonstrates the wide variety in sensations that are perceived from two types of PBCS. The combination of rating specific sensory and quality attributes along with liking ratings can provide a greater understanding of the product profile and the relationship each attribute to the overall sensory experience, either leading to acceptance or rejection.

## 4. Review of the Sensory Methods

Sensory preferences are like a fingerprint, unique to each individual and influenced by many factors but the pulse is fueled by the same source, where consumers’ purchase intention is ultimately driven by taste [32]. Regardless of whether the PBCS, either embraces the natural sensory characteristics of plant-based ingredients or closely mimics conventional dairy products, the finished product should have physical, functional, and sensory properties that consumers find desirable. However, as an instrumental technique, following proper standard protocols helps to increase rigor and minimize bias. While there is established work in PBCS using sensory approaches, common errors in sensory methodology and limitations of the current literature still exist. Below, we highlight some of these errors and limitations and also highlight areas that describe additional considerations when performing sensory evaluation for PBCS.

### 4.1. Limitations and Considerations for Current Literature

In the reviewed literature, there were differences in methodologies used, including data collection measures, number of participants, and whether participants received training. Many of the studies reported using trained panels which are known to induce bias when collecting hedonic ratings and may not be representative of the consumer population. While the incorporation of sensory analysis is helpful and suggests that it is a beneficial tool, there is an opportunity to improve upon the sensory methods utilized. There are limitations within the current literature that are notable, suggesting additional work is needed to validate findings. As noted above, the literature repeatedly describes beany flavor and gritty mouthfeel to be “off characteristics” and undesirable in soy products. These studies suggest that modifying fermentation, processing, or blending other plant-based milks approaches improves these sensory characteristics [16,17,18,19,20,21,22,23,24,25,26,27,28,29,30,31]. It is described that beany and gritty sensory attributes are reduced; however, no study directly measured these attributes during sensory analysis. To better understand the impact of blending milks and modifying processing steps of PBCS, sensory methods that quantify attributes, such as descriptive sensory analysis, could be performed to determine how these strategies impact sensory attributes such as beany and gritty. Other sensory qualities, such as mouthfeel characteristics, were understudied among these articles and provides an opportunity for future investigation of PBCS. Furthermore, out of the 12 studies reviewed, four did not undergo statistical analysis, and they were, therefore, unable to determine whether a significant improvement was achieved.

It should be noted that a combination of sensory methods can provide a more in-depth understanding between the perception of sensory attributes and their impact on liking/disliking. Integrating hedonic, discrimination, and/or descriptive testing can reveal important relationships between the sensory profile and consumer liking [33]. Another advantage is the combination of instrumental analysis with consumer liking. This approach can help to identify the connections between physical attributes with improved liking. For example, several studies reviewed here measured the textural profile which can help to link attributes like hardness and chewiness with consumer ratings of mouthfeel attributes; however, no formal analysis was conducted to better determine how textural attributes affected liking. Future studies can benefit from combining both sensory methods with instrumental analysis to provide a greater understanding of the physical attributes that drive liking and disliking of PBCS.

### 4.2. Consumers and Future Considerations for Segmentation

It is important to recruit a large and diverse group of participants screened based on consumption of PBCS or dairy-based cheese. Although smaller pools (*n*~20) are sufficient enough for trial hedonic testing, much larger groups (*n* = 75–150 [33] and even larger *n* = 200–500 [34,35] are crucial for more accurately predicting consumer acceptance in the market. While this process seems straightforward, recruitment for large sampling can be challenging due to screening parameters (i.e., allergies, availability, and product usage) and additional incurred costs, yet, provide a more rigorous/confident response rate. However, screening can provide a better understanding and deeper connection to types of PBCS consumers.

Dairy alternatives were once geared primarily toward consumers who actively avoided dairy due to allergy, intolerance, or a vegan diet. However, this is quickly changing due to emerging plant-based proteins and sensory quality advancements, coupled with concerns over environmental impacts, sustainability, health, and animal welfare, where more adults across dietary spectrums are choosing dairy substitutes [10]. In terms of PBCS and the current market, there are consumers who want to enjoy the nuance and embrace the uniqueness of the plant sensory in PBCS. Jeske et al. [5] explains that a noteworthy approach from manufacturers and consumers would be to appreciate the flavor of plant ingredients [5]. After all, why would a sunflower seed PBCS product not have a flavor profile of sunflower seeds [5]? Yet, there are consumers who expect PBCS to resemble their dairy counterparts in terms of traditional chemical (flavor, taste, and aroma) and physical (meltability and mouthfeel) properties. If the flavor, mouthfeel, or other sensory qualities of the end product are not what the consumer expects, it may result in rejection of that product [36]. With the potential for such a strong consumer segmentation, in the future, it may be worthwhile to further understand the diverse consumer segments of plant-based products. Consumers may also differ in whether the nutritional value of PBCS will influence acceptance, considering the differences in protein quality and calcium content, among other nutritional components, compared to conventional cheese products. If we better understand these consumer categories, it can be used to optimize plant-based materials specifically in PBCS to best cater to these different consumers.

Identifying an appropriate control is paramount for managing expectations and the performance of PBCS. Ultimately, the choice of the control hinges upon the goal of the PBCS, whether it is to embrace the uniqueness of the plant-based milk or to mimic sensory attributes of a dairy counterpart coupled with an all-encompassing consideration of sensory attributes, style, and functionality. Although the underlying goal of the PBCS was not explicitly expressed to the reader in the studies mentioned, it was gathered that there is a link between the goal and the choice of the control used in each study. While most studies used a 100% plant-based milk substitute as a control, three studies used a dairy counterpart [10,16,17]. If the intention is to embrace the natural PBCS attributes, comparing different kinds of PBCS will indicate if further refinement is needed or if the current product can be marketed. On the other hand, if the goal is to create a PBCS that fully mimics the chemical and physical attributes of conventional cheese then a dairy control should be used. Results will determine how the PBCS sensory properties compare to the dairy-based cheese product and depict whether further refinement is necessary or if there was an improvement in the development.

## 5. Conclusions

A human’s sensory perception is indispensable and the investment in sensory evaluation is imperative. Sensory science is an important part of a product’s development in its pre-commercial production life as it acts as the link in the chain which connects producers to consumers. Not only do consumers sometimes have the ability to detect odorants, among other sensory attributes, at lower levels than those of an instrument, but instruments cannot gauge pleasure, or predict liking as humans do [37]. Through the use of sensory evaluation, these studies demonstrate several promising processing techniques that can improve the overall quality (taste and textural) properties leading to increased liking of soy-based PBCS. These studies suggest that modifications in processing and addition of alternative milk products can improve consumer liking.

Emerging plant proteins may help to enhance the quality of PBCS. This can only be determined with the integration of sensory science. In terms of sensory evaluation, the only PBCS to be formally evaluated is limited to products either partially or fully prepared with soy and limited to spreadable products. Although some approaches reviewed here include blending nut milks with soy there are other plant-based ingredients that are used to produce PBCS without the use of soy. Few available studies explore other ingredients (i.e., corn [38], or zein [39] but do not incorporate sensory evaluation. Future research will help to uncover additional strategies along with more diverse plant-based ingredients to increase consumer acceptance of PBCS.

Sensory science can complement ongoing scientific research regarding PBCS in order to directly improve the quality attributes which can lead to more efficient development processes. Through the integration of sensory evaluation, product developers can gain a better understanding of ways to increase liking and acceptance by refining the sensory characteristics (i.e., flavor, meltability, mouthfeel, and aroma). Regardless of whether the PBCS intends to mimic the sensory experience of dairy-based cheese or embrace the naturally occurring flavors of plant-derived PBCS, expanding the product selection will better accommodate a broader audience of consumers which will therefore increase consumer sales.

## Figures and Tables

**Table 1:** Overview of PBCS articles employing sensory analysis.

Strategy	Ingredient	Sensory Method	Described Sample Size	Solution	Reference
Modified Fermentation	soy	H/D	10 panelists	Ferm/SB	Li, Q. et al., 2013 [16]
	soy	H/D	10 panelists	Ferm/SB	Li, Y. et al., 2020 [18]
	soy	H/D	14 participants	Ferm	Chumchuere et al., 2020 [19]
Blending Milks	soy/coconut	Hedonic	10 participants	Ferm/BM	Adejuyitan et al., 2014 [20]
	soy/groundnut	Hedonic	10 panelists	Ferm/BM	Khodke et al., 2014 [21]
	soy/tigernut	Hedonic	20 participants	Ferm/BM	Balogun et al., 2005 [22]
	soy	Hedonic	20 panelists	B*	Butool et al., 2015 [17]
Modified Processing	soy/coconut	Hedonic	not reported	Blanching/BM/SB	Kadbhane et al., 2019 [23]
	soy	Hedonic	20 participants	Acidification	James et al., 2016 [24]
	soy/cashew	Hedonic	30 participants	Blanching/BM	Oyeyinka et al., 2019 [25]
	soy/almond	Hedonic	50 participants	Ferm/BM	Arise et al., 2020 [26]
Commercial Products^A^	coconut	H/D	4 panelists	N/A	Saraco et al., 2020 [10]

H/D: hedonic and descriptive sensory methods; Ferm: fermentation; BM: blending milks; B*: blended carrot puree; SB: sodium bicarbonate; N/A: Compared commercially available products, Commercial Products^A^: One study compared attributes across commercially available products.

## Data Availability

Not applicable.
